# Effect of diabetic retinopathy and diabetes on the intraocular straylight in pseudophakic eyes

**DOI:** 10.1186/s12886-015-0120-1

**Published:** 2015-10-13

**Authors:** Hyung Bin Hwang, Hye Bin Yim, Sung Kun Chung

**Affiliations:** Department of Ophthalmology and Visual Science, Incheon St. Mary’s Hospital, College of Medicine, The Catholic University of Korea, Incheon, South Korea; Postal address: Department of Ophthalmology and Visual Science, St. Paul’s Hospital, College of Medicine, The Catholic University of Korea, 222 Banpo-daero, Seocho-gu, Seoul, 137-701 Republic of Korea

**Keywords:** Intraocular straylight, Diabetes mellitus, Pseudophakia

## Abstract

**Background:**

The aim of this study was to prove the relationship between the intraocular straylight level and diabetic retinopathy (DR) according to disease severity. Also, we aimed to evaluate whether diabetes mellitus (DM) per se could be a risk factor of increased intraocular straylight although we did not rely on a definite sign of DR in this study.

**Methods:**

In this prospective comparative study, ninety three eyes were enrolled and divided into four groups as follows: Group 1 (26 eyes), without DR or DM; Group 2 (25 eyes), with DM but without DR; Group 3 (21 eyes), mild to moderate non-proliferative DR; and Group 4 (21 eyes), severe non-proliferative DR. To measure the intraocular straylight in an objective manner, the C-quant straylight meter was used to preoperatively and 2 months postoperatively in all patients who underwent phacoemulsification surgery. All the patients also underwent a macular optical coherence tomography and hemoglobin A1c (HbA1c) analysis. A comparison of straylight levels adjusted by age among four groups was performed postoperatively.

**Results:**

The postoperative level of intraocular straylight was statistically significantly different among four groups (*P* <0.05). When adjusted for ages, Group 4 showed the highest straylight level when compared with Group 3 and the other two groups (*P* <0.05). Group 1 showed the lowest straylight level in comparison with Group 2 and the other two groups (*P* <0.05). There was no significant correlation between HbA1c level, duration of diabetes and postoperative straylight level.

**Conclusions:**

The level of intraocular straylight at 2 months postoperatively had a tendency to increase as the severity of DR increased. Additionally, the straylight level was higher in DM patients without DR than in patients without DM. Therefore, the severity of DR seemed to influence the intraocular straylight level. Although there is no definite sign of DR, DM per se can be a risk factor for increasing intraocular straylight. In conclusion, the level of intraocular straylight seems to be a sensitive test for detecting early retinal damage secondary to DM.

## Background

In an ideal eye, light scattering should be absent. Because the optical media of the human eye is not ideal optically, a certain amount of light scattering always occurs. This light scattering is called intraocular straylight [1]. Patients with straylight complain of the following symptoms: increased glare sensitivity, reduced ability to perceive contrasts in light or color, and haziness of vision. Thus, intraocular straylight can decrease the amount of contrast between the perceived image of an object and the surrounding image. Based on the compensation comparison method, a straylight meter (Oculus C-Quant, Oculus GmbH) can be used to determine the level of intraocular straylight. In this device, absolute straylight level is expressed as log (straylight parameter), or log(s).

Within the normal eye, the following five ocular structures contribute to the intraocular straylight: the cornea, iris, sclera, lens and retinal fundus [2]. Optical clarity is a fundamental property of the cornea. Therefore, corneal haze with decreased corneal transparency is related to increased light scattering [3]. Corneal factors such as corneal edema [4], dry eye [3] and corneal refractive surgery [5, 6], are associated with changes in intraocular straylight. Additionally, factors associated with the crystalline lens [7] and intraocular lens (IOL) [8, 9] are related with changes in straylight. However, there is a lack of studies demonstrating the effect of retinal disorders on intraocular straylight. Some studies reported that retinal diseases such as retinitis pigmentosa and choroideremia could affect the intraocular straylight [10–12]. However, these studies concluded that increased straylight in these retinal diseases was associated with early structural changes in the crystalline lens before the development of a clinically discernible posterior subcapsular opacity [12]. As far as we know, no previous study has described the relationship between intraocular straylight and pure retinal disorder.

In diabetic retinopathy (DR), contrast sensitivity (CS) function is reduced; thus, the CS test has been proven useful for diagnosing early and advanced DR [13–15]. Besides, CS is known to be significantly impaired in patients with diabetes mellitus (DM) without definite DR. Therefore, Katz et al. [16] suggested that the CS test can be an important tool for detecting early changes before overt DR occurs. They described that this impairment can be caused by neuronal damage at the level of photoreceptors before expressing clinically detectable microangiopathy in the retina of diabetic patients. As mentioned above, intraocular straylight can decrease the contrast of perceived images that fall on the retina. Based on the above, we hypothesized that retinal vascular disorders such as DR can also increase the intraocular straylight level.

The aim of the present study was to prove the relationship between the intraocular straylight level and DR according to disease severity. Additionally, we aimed to evaluate whether DM per se could be a risk factor of increased intraocular straylight although we did not rely on a definite sign of DR in this study.

## Methods

### Subjects

This prospective study involved eyes with cataracts planned to undergo routine phacoemulsification and posterior chamber IOL implantation between March 2012 and June 2014. In total, 67 eyes of 67 type 2 diabetic patients and 26 eyes of 26 non-diabetic patients were enrolled in this study. All the patients underwent detailed ophthalmologic examinations including measurement of best corrected visual acuity (BCVA), intraocular pressure, slit lamp examination of the anterior segment, fundus examination, central subfield macular thickness using optical coherence tomography (Stratus OCT, Carl Zeiss Meditec, Inc., Dublin, CA), and intraocular straylight. These examinations were implemented preoperatively and at 2 months postoperatively.

Medical data were collected for diabetic patients, including the duration of disease, comorbid medical disease such as hypertension and hyperlipidemia, fasting glucose and hemoglobin A1c (HbA1c) level. Ocular conditions that influence the intraocular straylight were excluded, including glaucoma, corneal opacities, and other retinal diseases such as definite macular edema on OCT and uveitis. Cataract surgery consisted of no-stitch, 2.8-mm clear corneal incision and phacoemulsification with implantation of an aspheric posterior chamber IOL (MI60, Bausch & Lomb, Rochester, NY, USA). This IOL is a hydrophilic acrylic, biconvex anterior and posterior aspheric lens with an aberration-free optic. All the enrolled patients in this study underwent implantation of this IOL with phacoemulsification. If the patient underwent cataract surgery on both eyes, we randomly chose and enrolled only one eye for this study. None of the patients presented any characteristics that would be expected to lead to an increased level of postoperative intraocular straylight because there were no specific complications during the cataract surgery or follow-up periods.

Before the enrollment in this study, all patients provided written informed consent in accordance with the protocol approved by the Helsinki Committee. Additionally, this study was approved by Institutional Review Board of the Catholic University of Korea.

### Division of groups

In this prospective study, 93 eyes were enrolled and divided into four groups. Patients were graded for the presence or absence and severity of DR according to the findings observable upon dilated ophthalmoscopy as shown in Table 1. The four groups were as follows: 26 patients (26 eyes) in Group 1 without DM or DR, 25 patients (25 eyes) in Group 2 with DM but without DR, 21 diabetic patients (21 eyes) with mild to moderate non-proliferative diabetic retinopathy (NPDR) in Group 3, and 21 diabetic patients (21 eyes) with severe NPDR in Group 4. None of the patients presented obvious diabetic macular edema because there was no apparent retinal thickening or hard exudates in the posterior pole in the fundus examination and macular OCT analysis.

**Table 1:** Diabetic retinopathy disease severity scale according to the Early Treatment Diabetic Retinopathy Study (ETDRS)

*Mild non-proliferative diabetic retinopathy*	
Microaneurysms only	
*Moderate non-proliferative diabetic retinopathy*	
More than just microaneurysms but less than severe non-proliferative diabetic retinopathy	
*Severe non-proliferative diabetic retinopathy*	
Any of the following:	
More than 20 intraretinal hemorrhages in each of four quadrants	
Definite venous beading in more than two quadrants	
Prominent intraretinal microvascular abnormalities in more than one quadrant and no signs of proliferative retinopathy	

### Measurement of intraocular straylight

Based on the compensation comparison, a straylight meter (Oculus C-Quant, Oculus GmbH) was used to measure the level of intraocular straylight (The Netherlands Royal Academy has a patent with Thomas Van den Berg inventor licensed to Oculus GmbH for the C-quant straylight meter). That is, the straylight meter was used to objectively measure intraocular straylight preoperatively and 2 months postoperatively after all patients underwent phacoemulsification surgery. Briefly, the C-quant measures the level of intraocular straylight by compensating for the amount of straylight on a test field, with counter-phase flickering of the test field of variable intensity. The intensity of the counter-phase flickering required to compensate for the induced flickering by the straylight is a measure of the level of intraocular straylight [17]. Before each measurement, the refractive correction was carried out, if necessary. The C-quant straylight meter took measurements in photopic conditions, and measurements were taken in complete darkness to avoid any light from other sources [18]. When the measurement was completed, the reliability of the result was evaluated. The measurement was considered reliable when standard deviation of the measured straylight value (Esd) ≤0.08 and reliability coefficient (Q) ≥1. Intraocular straylight measured in these enrolled patients was thought to be caused by the DR only because the effect of lens opacity could be excluded in these pseudophakic eyes.

### Statistical analysis

All data were analyzed using SPSS 12.0 for Windows statistical software (SPSS, Inc., Chicago, IL, USA), and expressed as the mean ± standard error (SE). After the cataract surgery, the BCVA (expressed in a logMAR scale), HbA1c level, and central subfield macular thickness among the four groups were analyzed using one-way analysis of variance (ANOVA). The intraocular straylight level among the groups was analyzed using analysis of covariance (ANCOVA) for controlling of bias factors, such as age. For pairwise comparisons, the Bonferroni test was used for *post-hoc* analysis, assuming equal variance. Additionally, regression equations were calculated between intraocular straylight and age using regression analysis. A *P* value of <0.05 was considered statistically significant.

## Results

Baseline characteristics of the patients in the four groups are described in Table 2. There was no statistically significant difference in preoperative BCVA and preoperative intraocular straylight level among the four groups (*P* = 0.155, 0.202 respectively, one way ANOVA). Because we enrolled patients without macular edema in this study, there was no statistically significant difference central subfield macular thickness among four groups (*P* = 0.128, one way ANOVA): 222.77 ± 2.74 μm, 217.36 ± 2.06 μm, 218.14 ± 2.78 μm, and 225.43 ± 3.28 μm as mean ± SE.

**Table 2:** Baseline characteristics of the four groups

	Group 1 (26 eyes)	Group 2 (25 eyes)	Group 3 (21 eyes)	Group 4 (21 eyes)
Age (years)	68.96 ± 1.40 (55–79)	71.40 ± 1.18 (57–82)	60.91 ± 1.61 (45–73)	63.95 ± 1.80 (51–77)
HbA1c (%)	-	6.67 ± 0.14 (5.90–8.20)	6.40 ± 0.13 (5.20–8.10)	6.99 ± 0.18 (5.80–8.70)
Duration of DM (years)	-	22.69 ± 1.18 (13–34)	13.86 ± 1.02 (5–21)	16.10 ± 0.87 (8–23)
Central macular thickness (μm)	222.77 ± 2.74 (189–242)	217.36 ± 2.06 (197–241)	218.14 ± 2.78 (198–242)	225.43 ± 3.28 (198–250)
Preoperative straylight (log(s))	2.08 ± 0.05 (1.56–2.37)	2.18 ± 0.04 (1.65–2.35)	2.19 ± 0.04 (1.48–2.36)	2.13 ± 0.04 (1.56–2.36)
Preoperative BCVA (LogMAR)	0.48 ± 0.04 (0.20–0.90)	0.48 ± 0.03 (0.30–0.70)	0.56 ± 0.05 (0.20–1.00)	0.58 ± 0.04 (0.30–1.00)
Postoperative straylight (log(s))	1.21 ± 0.04 (0.68–1.43)	1.44 ± 0.06 (1.14–2.03)	1.45 ± 0.03 (1.19–1.69)	1.70 ± 0.09 (1.16–2.24)
Postoperative BCVA (LogMAR)	0.08 ± 0.01 (0.00–0.18)	0.10 ± 0.01 (0.00–0.18)	0.10 ± 0.02 (0.00–0.18)	0.14 ± 0.02 (0.00–0.30)

Data in the table are presented as means ± standard errors

*HbA1c* = hemoglobin A1c, *DM* = diabetes mellitus, *BCVA* = best corrected visual acuity

**Fig. 1:**
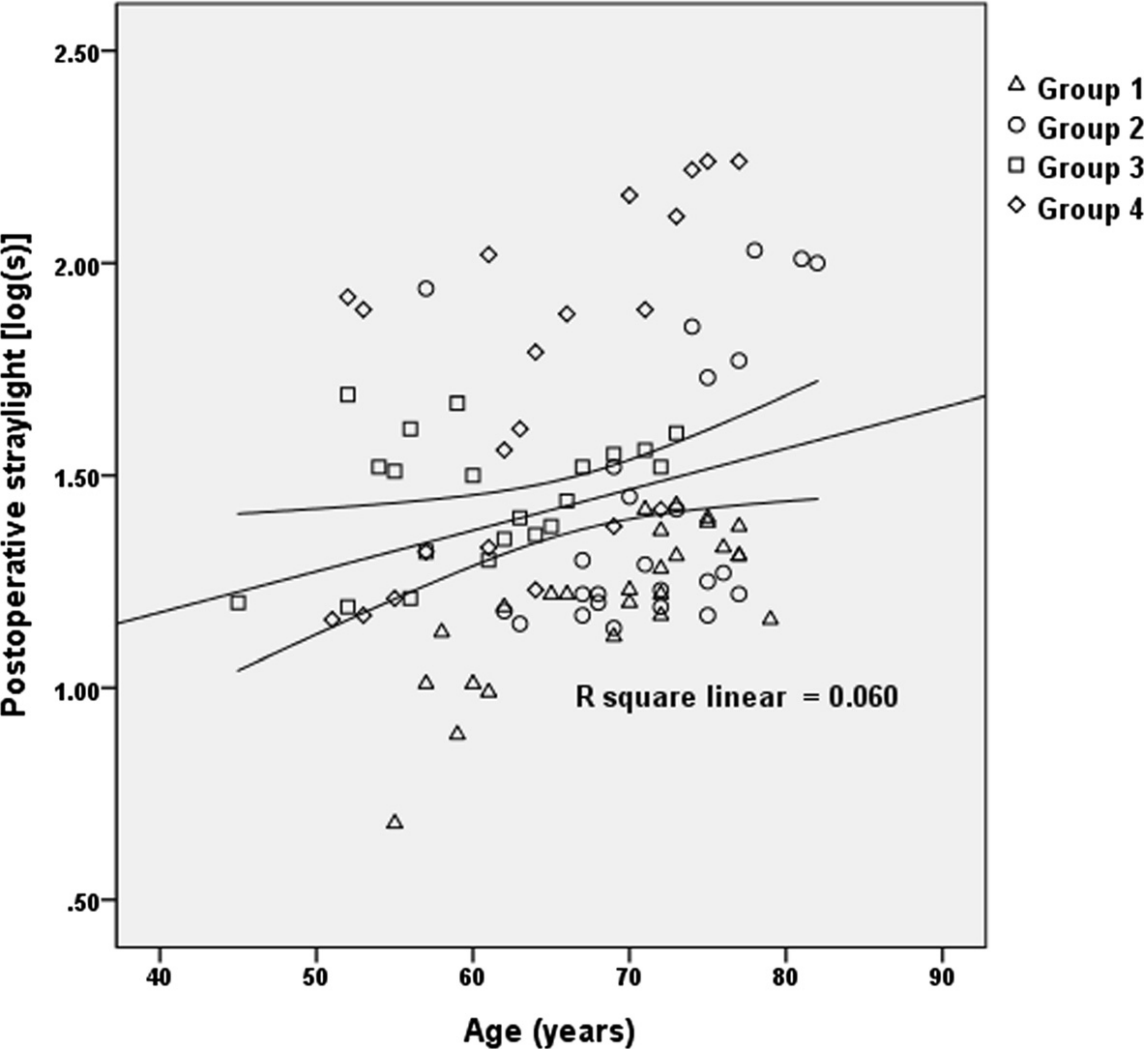
Linear regression analysis of the measured straylight levels in the four groups. The regression analysis showed a weak relationship between age and straylight level. The equation was as follows: log(s) = 0.790 + age × 0.010 (*P* = 0.018, R^2^ = 0.060)

Before comparing the values of postoperative straylight among the four groups, we evaluated the factors of covariates that influenced the straylight level using tests of between-subjects effects in ANCOVA. As a result, HbA1c, duration of diabetes and central macular thickness showed no significant result, and age was the only covariate that influenced the postoperative intraocular straylight level (*P* = 0.540, 0.569, 0.884, and 0.001, respectively, ANCOVA, data not shown). As shown in a previous study [8], there was a weak positive correlation between age and intraocular straylight in our pseudophakic eyes (Fig. 1). After adjusting for age, the partial correlation analysis, between HbA1c and postoperative straylight level was not significant (r = 0.209, *P* = 0.092, partial correlation test, data not shown). Additionally, there was no correlation between the duration of diabetes and postoperative straylight level when adjusting for age (r = −0.236, *P* = 0.056, partial correlation test, data not shown). That is, there was no significant correlation between HbA1c level, duration of diabetes and postoperative straylight level.

**Fig. 2:**
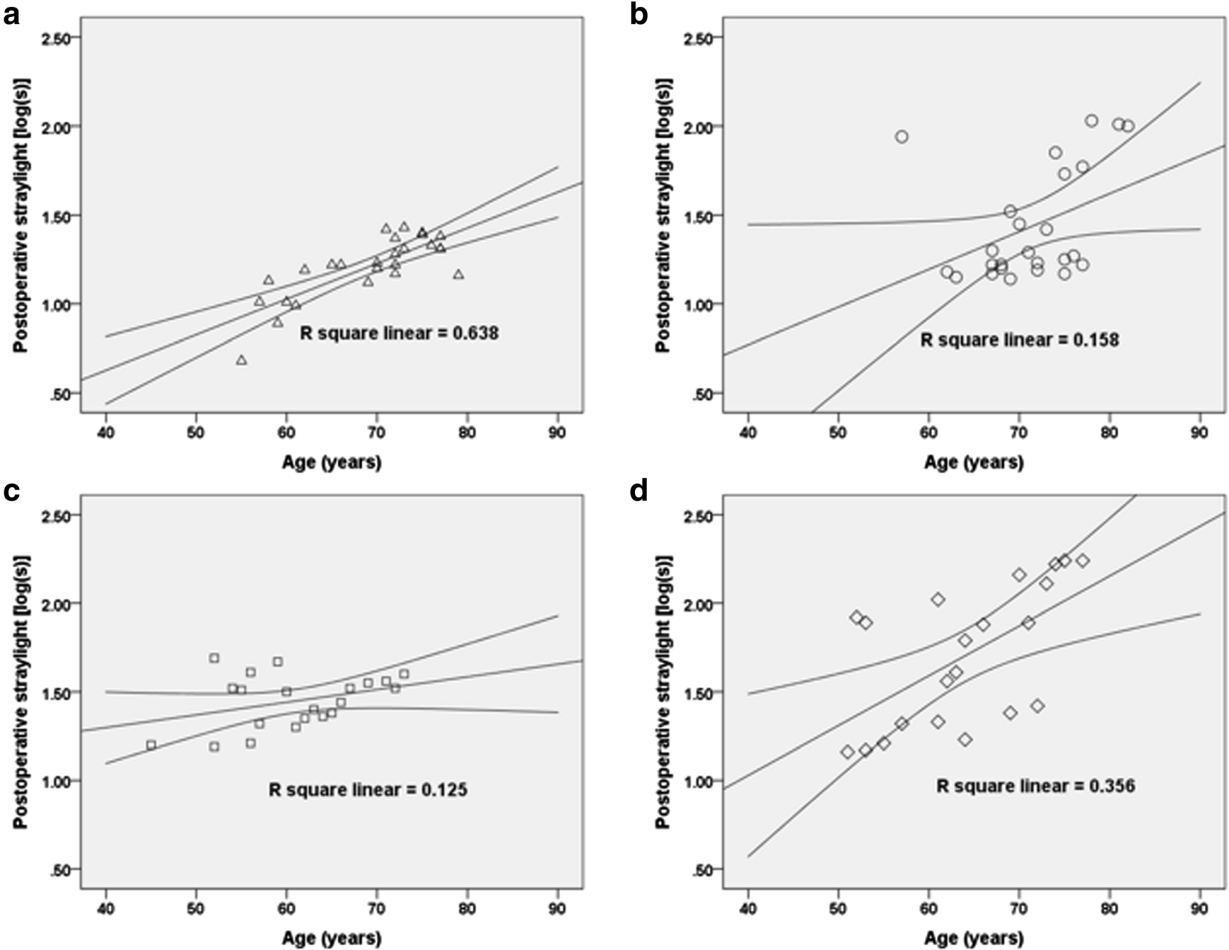
Linear regression analysis of measured straylight levels according to each group (equations in Table 3). **a**. Group 1, **b**. Group 2, **c**. Group 3, **d**. Group 4

The value of estimated marginal means of postoperative intraocular straylight which was corrected for differences in age between subgroups showed a significant difference among the four groups (*P* <0.001, ANCOVA). The means and linear regression equation are demonstrated in Table 3 and Fig. 2. The *post hoc* analysis was conducted among the four groups by implementing pairwise comparison using Bonferroni analysis. As a result, Group 4 showed the highest level of postoperative straylight compared with the other three groups. Group 3 showed highest straylight level when compared with Groups 1 and 2. Group 2, that is, DM patients without DR showed higher straylight level than Group 1, that is, patients without DM (Table 4).

**Table 3:** Estimated marginal means adjusted by age with linear regression equation

	Group 1	Group 2	Group 3	Group 4
Estimated marginal means	1.162 ± 0.047	1.344 ± 0.050	1.560 ± 0.055	1.756 ± 0.053
95 % confidence interval	1.068–1.255	1.244–1.444	1.450–1.671	1.651–1.860
Multiple linear regression	log(s) = −0.174 + age × 0.020	log(s) = −0.079 + age × 0.021	log(s) = 1.011 + age × 0.007	log(s) = −0.097 + age × 0.028

**Table 4:** Pairwise comparison among the four groups using Bonferroni analysis

Group	Estimated marginal means of log(s)	Pairwise comparison with the other groups	*P* value
1	1.162 ± 0.047	2	0.021
		3	0.021
		4	<0.001
2	1.344 ± 0.050	1	0.021
		3	0.046
		4	0.010
3	1.560 ± 0.055	1	0.021
		2	0.046
		4	0.020
4	1.756 ± 0.053	1	<0.001
		2	0.010
		3	0.020

## Discussion

The natural human crystalline lens and iris can be important sources of light scattering [2]. Therefore, if the crystalline lens is replaced with a clear IOL during cataract surgery, the new lens may become a source of light scattering. Assuming an IOL is made of nearly perfectly clear material, we could theoretically exclude the effect of lens opacity on light scattering. Intraocular straylight was measured with normal pupillary size of roughly 2 to 7 mm in diameter under low photopic conditions, to exclude the effect of the iris on the amount of straylight measured. Additionally, straylight is strongly correlated with age and is known to increases rapidly after the age of 50 years in the normal population [19]. Thus, we analyzed the effect of DR on the straylight using the ANCOVA test to exclude the bias of age.

We found that a significant impairment of the intraocular straylight was revealed as the severity of DR increased in this study. Interestingly, this impairment of straylight was also applied in the diabetic patients even though they did not present definite DR in the test of dilated ophthalmoscopy. However, in our study, there was no correlation between intraocular straylight level and HbA1c. This means that the degree of glycemic control did not affect the amount of intraocular straylight. The Early Treatment Diabetic Retinopathy Study (ETDRS) identified important risk factors of the progression to high-risk proliferative diabetic retinopathy (PDR), including retinopathy severity, decreased visual acuity, high levels of HbA1c and serum lipids level [20]. That is, a poor control of DM influences the progression of DR. However, this point was not supported by our findings because there was no significant difference in HbA1c levels among Groups 2, 3, and 4. We assumed that this occurred because only patients with NPDR were enrolled in this study. If the patients with PDR and CSME would have been enrolled in this study, perhaps we could have detected a significant difference in the HbA1c level among the groups. According to our results, poor glycemic control may not contribute to the increase of intraocular straylight.

To the best of our knowledge, this study is the first to investigate the relationship between the severity of DR and the level of intraocular straylight. However, many studies have investigated the effect of DR on other visual functions, such as CS. Previous studies indicated that abnormal and reduced CS was observed in patients with DR more frequently than in patients without DR [14, 21]. These studies suggests that the measurement of CS could provide a clinical option for further identifying early DR in the patients with DM. Brinchmann-Hansen et al. also reported that CS of 6 cycles per degree was associated with the grade of retinopathy, and patients with retinopathy had reduced visual function compared to those without [22]. Consistent with the lack of correlation between straylight and HbA1c in this study, they concluded that reduced CS was not related to glycemic control, duration of diabetes or urinary albumin excretion. Katz et al.[16] suggested that mesopic foveal CS is impaired in diabetic patients even though they have a good visual acuity, normal fundus and normal OCT findings. We also concluded that intraocular straylight was increased in the DM patients without definite DR when compared with the control group. Therefore, we concluded that the measurement of intraocular straylight by C-quant could be a useful clinical screening tool for detecting early retinal change before overt DR occurs.

Of course, the cause of increased straylight in DR could not be limited to retinal factors. Diabetic keratopathy could be another complication of diabetes [23] that can cause increased straylight. Takahashi et al. [24] proved that light scattering at the corneal basement membrane increased proportionally with the extent of DR. Further, Morishige et al. [25] measured the light scattering of the corneal epithelial basement membrane in patients with type 2 DM with DR and healthy subjects and found that the light scattering index was significantly higher in patients with DM with PDR compared with healthy controls. Furthermore, patients with DM are known to experience vitreous degeneration earlier than those without DM [26]. Although no previous study has demonstrated the relationship between diabetic vitreopathy and light scattering, factors associated with the vitreous can affect the optical quality of the eye [27]. Future studies demonstrating the relationship between diabetic vitreopathy and intraocular straylight would be necessary.

We did not evaluate the pupil size before and after cataract surgery nor its effect on intraocular straylight. That is, pupil size was not taken into account in this study. Cataract extraction surgery is known to affect pupil size and centration [28, 29]. However, none of our patients had any pupillary abnormalities and their pupils were within the normal size range after the cataract surgery. In our study, straylight was likely to be measured in eyes with different pupil sizes. The C-quant was used under photopic luminance conditions, and thus, the pupil size always corresponded to photopic pupil size. However, Franssen et al. [17, 30] reported that in natural pupils approximately 2–7 mm in diameter, straylight can be regarded only as weakly dependent on the pupil size (within 0.02 log units). That is, in pupils within the normal size range, pupil size has little effect on straylight.

It is not unclear why the intraocular straylight is significantly increased in diabetic patients with DR and without definite DR compared with non-diabetic controls. According to the study by Lopes de Faria et al. [31], a significant and nonselective neuronal visual loss involving the visual pathway precedes the ophthalmoscopically detectable retinopathy in diabetic patients. Further, the retinal fundus is known to be a major contributor to the amount of intraocular straylight [2]. Thus, we hypothesized that this increment of straylight might be caused by neuronal damage at the level of the photoreceptors rather than to clinically detectable diabetic retinal microvasculopathy. However, further study would be necessary to evaluate whether the diabetic patients with increased straylight could be considered at high risk developing DR.

The correlation between intraocular straylight and CS still remains to be elucidated. Forward scattered light projects a veil of light over the retinal image that decreases the contrast of the image. This veil of light is called intraocular straylight [1, 32]. Therefore, increased straylight indicates a lower CS. However, the decrease in CS is much smaller than the increase in straylight; a 5-fold increase in light scattering lowers CS function by only 20 %. According to Ralph et al. [33], there is a weak correlation between CS and the amount of intraocular straylight. Nevertheless, a certain amount of correlation seems to exist between intraocular straylight and CS. Therefore, we hypothesized that measuring the straylight level could be a useful clinical screening tool for detecting early diabetic retinal changes alike the CS. Further research on larger cohorts should be conducted to investigate the role of straylight measurement in DR screening.

## Conclusion

The level of intraocular straylight seems to be a sensitive test for detecting early retinal damage secondary to DM, in spite of good visual acuity, normal fundus finding and normal OCT findings. We consider that decreased CS and light scattering could occur in diabetic patients before the appearance of retinopathy. Regardless of the prognostic or diagnostic value of these tests, C-quant could be a suitable screening tool to detect early changes before overt DR occurs. Future studies should be conducted to establish the role of the straylight meter for the screening DM.
